# Numerical Simulation of Laser Transmission Welding—A Review on Temperature Field, Stress Field, Melt Flow Field, and Thermal Degradation

**DOI:** 10.3390/polym15092125

**Published:** 2023-04-29

**Authors:** Shuangxi Hu, Fang Li, Pei Zuo

**Affiliations:** 1 Hubei Provincial Key Laboratory of Chemical Equipment Intensification and Intrinsic Safety, School of Mechanical and Electrical Engineering, Wuhan Institute of Technology, Wuhan 430205, China; 2 School of Mechanical and Electrical Engineering, Hubei Open University, Wuhan 430070, China

**Keywords:** laser transmission welding, numerical simulation, temperature field, residual stresses, melt flow field

## Abstract

Laser transmission welding (LTW) is an excellent process for joining plastics and is widely used in industry. Numerical simulation is an important method and area for studying LTW. It can effectively shorten the experimental time and reduce research costs, aid in understanding the welding mechanism, and enable the acquisition of ideal process parameters. To enhance understanding of numerical simulation studies on LTW and facilitate research in this area, this paper presents a comprehensive overview of the progress made in numerical simulation of LTW, covering the following aspects: (a) characteristics of the three heat source models for LTW temperature field simulation, including surface heat source model, volumetric heat source model, and hybrid heat source model, along with the methods, results, and applications of temperature field simulation based on these models and experimental validation; (b) numerical simulation of thermal and residual stresses based on the temperature field; (c) numerical simulation of the melt flow field; and (d) predictive simulation of material degradation. The conclusion of the review and the prospects for further research work are eventually addressed.

## 1. Introduction

Laser transmission welding (LTW) is a non-contact, high-efficiency, non-polluting, and flexible plastic joining technology. It offers superior advantages over traditional methods such as friction welding, ultrasonic welding, and resistance welding. Due to these advantages, LTW has a wide range of applications in various industries, such as automotive, aerospace, medical consumables, and food packaging [1,2,3,4,5].

**Figure 1:**
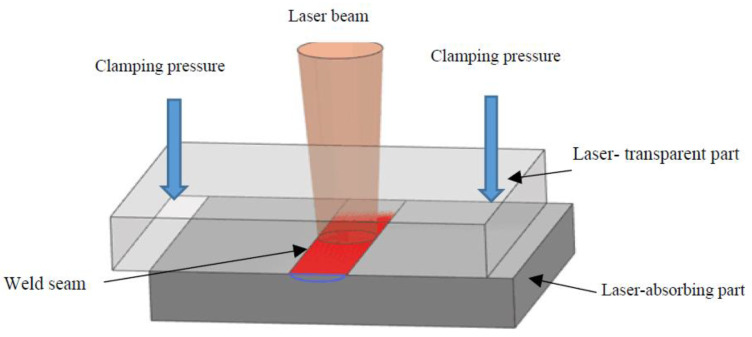
Schematic diagram of the plastic laser transmission welding principle.

In the LTW process, the two parts to be joined must have sufficient weldability, meaning that they should be able to be welded together using a laser. Then two plastic parts are lapped together, and an external clamping force is applied to make a tight fit between the two parts, as shown in Figure 1. The top part is highly transparent to the laser, and the bottom part is highly absorbent of laser energy [6]. The high-energy laser beam passes through the top part, is absorbed by the bottom part and transformed into heat in a very short time, and then conducts heat to the top part. The temperature between the interfaces rises rapidly, causing the materials of the two parts to melt, spread, and form a weld under both the clamping pressure and thermal expansion. When the bottom part has low laser absorption capacity, it is common practice to enhance its absorption of laser energy by coating its surface with absorbers, such as carbon black or metal particles [7], or by mixing and injecting absorbers into the bottom part.

LTW includes contour welding, quasi-synchronous welding, synchronous welding, mask welding, radial welding, etc. The materials used for LTW are generally remeltable thermoplastic materials. Continuous-wave infrared radiation with a wavelength of 0.8–1.1 μm is usually used [8]. Weld appearance, dimensions, and joint strength are important representations of the quality of welded joints. In laser transmission welding, if the heat input is too high, the joint may be degraded due to excessive heat, which can result in reduced joint strength. On the other hand, if the heat input is too low, the joint may not be completely fused, also leading to low joint strength. The uneven heating of the welded parts can result in stress cracking and the formation of bubbles [9], both of which can adversely affect the quality of the welded joint. The welding temperature plays a crucial role in determining the quality of the weld, and it depends on various factors, such as the nature and composition of the welding polymer and absorber, the laser system, the welding strategy [10], etc.

The reflection, transmission, and absorption of laser light are affected by the pigment, filler, and additive status of the polymer [11,12,13], which in turn affects the absorption of laser energy for welding. Parameters such as laser power, scanning speed, and beam diameter affect the input of this laser energy [14,15]. Welding strategies such as beam shape and wavelength also affect the distribution and absorption of laser energy, ultimately impacting the quality of the weld.

A large body of literature has studied the composition of polymers in LTW, including the influence of absorbers on weld quality [16,17,18,19], different transparent materials, cases of high scattering materials [20,21], and welding of polymers and metals [22,23,24]. To improve joint strength and quality, numerous studies have been conducted to determine the optimal welding parameters, such as welding power, scanning speed, absorber content, etc. Additionally, researchers have explored alternative welding strategies for LTW, including changing the wavelength and beam shape and adopting oscillating welding methods [25]. The above studies not only relied on experiments but also employed numerical simulation as an approach.

The introduction of numerical simulation can effectively reduce the cost of experiments, shorten the process experiment time [26], efficiently obtain the ideal process parameters, and improve the quality of welding. Numerical simulations provide a theoretical–simulation-production basis for LTW technology and play an important role in the study of laser welding theory and processes. Here, numerical simulations are mainly used to study the effect of different welding materials and process parameters on the temperature field, melt flow field, and stress field, both individually and in combination. This analysis helps to understand the influence of each factor on the welding quality and to explore the connection mechanism and optimal laser welding process. This paper provides an overview of the numerical simulation of LTW, including temperature field simulation using various heat source models of LTW along with experimental validation, stress field simulation and experimentation, flow field simulation and validation, and thermal degradation simulation. The purpose is to provide an in-depth introduction to the welding mechanism and process parameters of LTW.

## 2. Temperature Field Numerical Simulation of LTW

Temperature change is the fundamental cause of welding quality in the LTW process, so the temperature field is an important aspect of LTW research. Numerical simulation, especially through the use of commercial software such as ANSYS and COMSOL, is commonly employed to investigate the temperature field in LTW research. In the numerical simulation of the temperature field, the research idea follows the following route: determining the type of heat source model → determining the heat source parameters and boundary conditions → numerical simulation → test comparison verification [27].

The welding heat source model is fundamental to the numerical simulation of the welding process [28,29]. The accuracy of the heat source model determines the accuracy of the finite element calculation results of the welding temperature field. In LTW, the surface heat source model, the volume heat source model, and the combined heat source models are mainly used for numerical analysis.

The planar heat source model describes the surface distribution of the heat source, which is suitable for cases where the thickness of the weld is not large and the melt pool is shallow. However, it cannot express the temperature distribution in the depth direction. The bulk heat source model describes the distribution of the heat source in the melt depth direction and has high computer accuracy. This heat source model is suitable for narrow and deep melt pools generated by high-energy welding methods such as laser welding. The combined heat source model requires selecting a suitable superposition of multiple heat source models, which involves difficulties such as parameters that are not easy to determine and a large computational effort. The combined heat source model is suitable for the simulation of complex welding conditions, such as composite welding technology.

Different models have different advantages and limitations. Therefore, several factors need to be considered when choosing the most suitable model for a specific LTW application, including the welding method, welding parameters, welding materials, and other relevant factors. For laser transmission welding, the form and content of the absorber material affect the distribution of heat sources. For instance, carbon black powder coated on the surface of the workpiece usually results in surface absorption, while carbon black uniformly injected into the material generally results in volume absorption. As the carbon black content increases, the energy absorption may change from volume absorption to surface absorption. Therefore, choosing an appropriate heat source model can not only better reflect the distribution pattern of the actual welding process but also reduce the calculation workload while ensuring the accuracy of the calculation. The numerical simulation study of different heat source models will be described in detail later in 2.1–2.4.

During the welding process, the bottom part absorbs laser energy for heat transfer. There are three main basic forms of heat transfer in LTW: heat conduction, convection, and thermal radiation. In numerical simulations, a moving heat source is generally used [30], and the coordinates that describe its position are related not only to the spatial coordinates but also to the duration of the process. As the heat source moves, the temperature of the entire weld dramatically changes in time and space. Additionally, the density, specific heat capacity, and other thermophysical properties of the material also vary dramatically with temperature, making the analysis of the laser welding temperature field a typical nonlinear transient heat transfer problem. The transient equation of the temperature field in the material is obtained based on the Fourier differential equation [17].

(1) $$\rho C_{p}\frac{\partial T}{\partial t}+\nabla \cdot （-K\nabla T）+h_{c}S\left(T-T_{ref}\right)\nabla \rho C_{p}T=Q$$

where ρ is the material density, *C_p_* is the specific heat capacity, *K* is the heat transfer coefficient, *h_c_* is the heat convection coefficient, *S* is the surface area of the weld, and *Q* is the heat source. 

During the welding process, various heat transfer mechanisms occur, such as convection and thermal radiation between the air and molten liquid at the interface of the workpiece. The contact area at the interface between the welded parts transfers heat to the upper parts through heat conduction. Due to the presence of gaps and air between the interfaces, the low-temperature air absorbs the heat from the surface of the lower parts when heated, and the temperature rises, which will transfer heat to the contact surface of the transparent parts. In the numerical simulation, the boundary conditions are set according to the heat transfer condition, and some assumptions and simplifications are often employed for the actual situation.

Due to the complexity of the laser transmission welding conditions and many influential factors, the numerical simulation results of the temperature field need to be calibrated and verified experimentally [31]. Validated measurement methods for LTW temperature fields include thermocouple methods, camera methods [15], and pyrometer methods [32]. The thermocouple method involves drilling multiple holes in the workpiece at different thicknesses and distances from the centerline of the weld and placing thermocouples to measure the temperature. This method is not very accurate because the laser energy is directly absorbed by the wire, which has high thermal conductivity and produces conduction errors. To measure the temperature of the weld seam when the upper part is transparent, visible light imaging is often used. This involves capturing images of the weld seam using a light-sensitive camera such as a CCD or CMOS [33]. Infrared cameras are used where the upper part is opaque to visible light but transparent to infrared radiation. Infrared thermography is applied to measure the temperature and the size of the heat-affected zone during LTW [3]. Pyrometers integrated into scanners are used for in situ monitoring of the laser welding process [33]. Since it is not easy to measure the temperature field accurately, the temperature field is generally verified indirectly by observing the weld morphology and measuring the HAZ dimensions of the weld.

The simulated temperature field results were used to further design the experiment [34,35]. The mathematical model between the process parameters and the welding results was established by using the response surface methodology (RSM) [36,37], artificial neuronal networks (ANN) [38], or some other methods. The relationship between input variables such as power, welding speed, and beam diameter and the output variables such as the maximum temperature of the weld interface, weld width and depth of the weld, and weld strength was obtained and verified using FEM simulation results. Intelligent simulation and optimization algorithms are integrated to obtain the optimal process parameters [23] and to find the optimal variables to improve welding quality and efficiency.

### 2.1. Temperature Field Numerical Simulation Based on Surface Heat Source Model 

**Figure 2:**
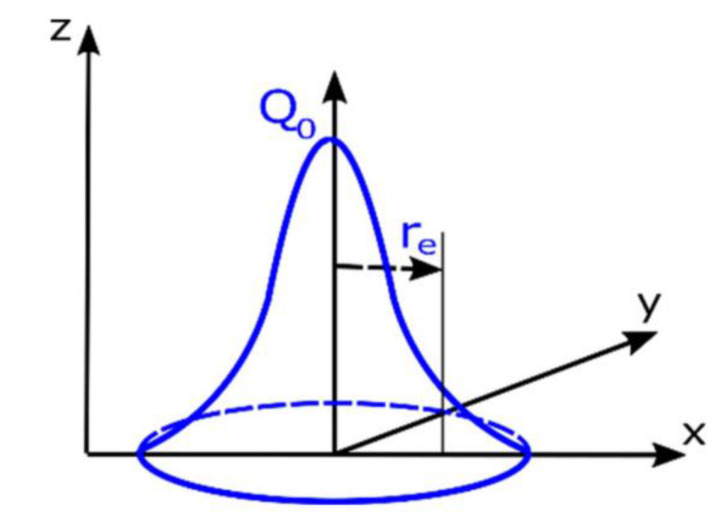
Gaussian surface heat source model [31].

The surface heat source model is suitable for welds with shallow melt depths and is divided into the planar Gaussian heat source model and the double elliptical heat source model. In the LTW process, the energy density distribution on the laser absorber assembly generally uses a planar Gaussian heat source model. The planar Gaussian surface heat source is a Gaussian function that distributes the energy of the heat source in a circle of a certain radius, with the maximum energy in the center of the heat source and decreasing from the center to the edge, as shown in Figure 2.

The distribution of heat sources based on plane Gaussian heat sources in LTW can be expressed as:

(2) $$I\left(x,y\right)=\left\{\begin{array}{c} \;\;0;\;for\;transparent\;part\\ \alpha \cdot \frac{P_{1}}{\pi R^{2}}\cdot \exp\left(-\frac{x^{2}+y^{2}}{R^{2}}\right);for\;absorbing\;part \end{array}\right.$$

where *P*_1_ corresponds to the power (W) when the laser reaches the surface of the absorption layer after passing through the transmission layer; α is the absorption layer coefficient; *R* is the spot radius (mm); and *x* and *y* are the point coordinates in the surface heat source. 

A finite element model of the LTW of PET and PP was established with a planar Gaussian heat source [39], and the temperature field distribution was simulated for laser power 0.9 W, welding speed 2 mm/s, and out-of-focus distance +2 mm. The maximum temperature during welding reached 318.796 °C, with the highest temperature not at the leading edge of the temperature profile but at the center or slightly later. The simulations revealed that the weld depth of the upper transparent PET layer was greater than the depth of the absorbing PP layer, unlike the general rule that the penetration depth of transparent polymers is less than that of absorbing polymers [37,40]. The specific reason for this was that the thermal conductivity of PET was greater than that of PP. The mathematical model established by the RSM predicts the values of weld width (W) and melt depth (D). The model indicates that the melt pool D/W ratio had a significant effect on the shear strength, which first gradually increased and then rapidly decreased as the D/W ratio increased.

An integrated approach combining finite element analysis (FEA), a kriging model, and the non-dominated sorting genetic algorithm II (NSGA-II) was used to obtain simulation data on the temperature field distribution and melt pool geometry for modeling and optimization mask-assisted laser transfer of microconnected thermoplastic polyurethane and PA6. The results of validation experiments under the optimal parameters showed that the corresponding welded joint quality was significantly better than under other parameters [41].

For the case where the absorber is located on the bottom surface, the numerical simulation mostly uses the surface heat source model. The welding heat is mainly concentrated near the absorber and conducted to the top part, so the simulation assumes that the heat is loaded only on the metal absorbers to load the Gaussian heat source. Chen et al. [42] established a 3D thermal model based on the surface heat source model for welding two transparent PMMA parts with a multicore copper wire as the absorber. According to the simulated and experimental data of the temperature, the absorption ratio of laser energy is 0.11 and 0.14 for single-core and multi-core copper wire, respectively. Compared to single-core copper wire, multi-core copper wire can reduce laser reflection and increase the laser energy utilization factor by 27.3%. In the study of the influence of the thermal conductivity of the metal absorber on the weld quality [43], a model of the LTW temperature field was established for four metals: titanium, nickel, molybdenum, and copper. The surface of the metal wire is divided into a limited number of small areas. The results show that when welding with an absorber with higher thermal conductivity, heat diffuses faster along the wire, resulting in a lower maximum temperature. This allows for more laser input power to be used, leading to a wider weld width and higher bonding force.

For LTW of plastics to metals, the metal has a strong absorption of laser energy, so most numerical simulations are performed using a surface heat source model. A three-dimensional finite element method was established to simulate the laser transmission joining of medical PET films with thin titanium plates, both 0.1 mm thick, using a Gaussian surface heat source [44]. The joint width was calculated for different process parameters, and the results showed that an increase in laser power or a decrease in welding speed increased the maximum temperature and joint width [17], which is consistent with the behavior observed in conventional LTW. Higher laser power and lower welding speeds result in partial decomposition or ablation at temperatures greater than the melting point of PET. Numerical simulations of the LTW of PET films on 304 L stainless steel [45], polyimide, and thin titanium plates [46] yielded the same results. Comparison with measured values of the test joint width verified the simulation results.

### 2.2. Temperature Field Numerical Simulation Based on Volume Heat Source Model

The Gaussian surface heat source model does not consider the influence of the heat source on the depth of the molten pool. In contrast, the volume heat source model is more accurate in modeling the welding temperature field and predicting the geometry of the weld pool, and it is suitable for high-energy welding methods such as laser welding, electron beam welding, and other scenarios that require high precision in calculating the welding temperature field.

**Figure 3:**
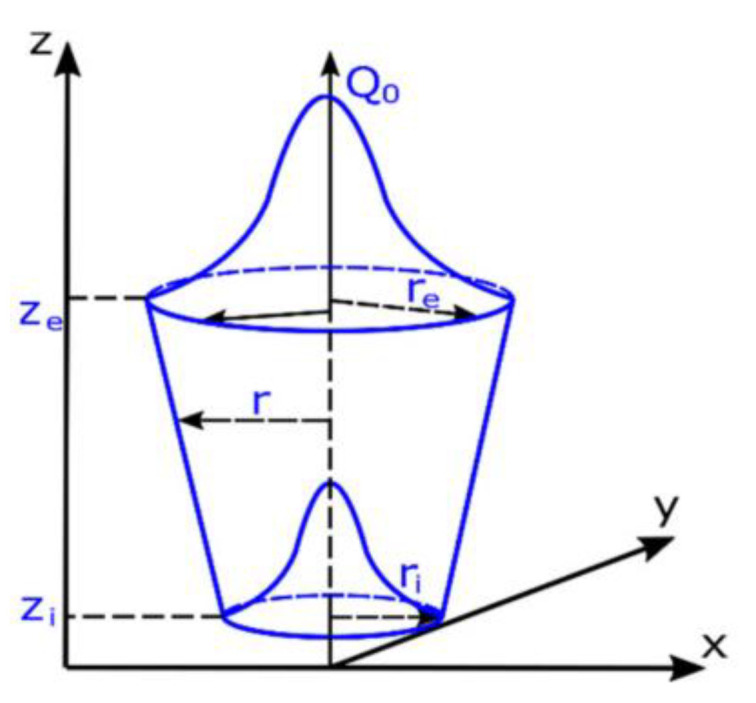
Rotating Gaussian volume heat source model [31].

The volume heat source model is divided into the uniform volume heat source model, the rotating Gaussian volume heat source model, and the double ellipsoidal volume heat source model [47]. The rotating Gaussian volume heat source model is used more often. John Goldak [48] proposed a rotating Gaussian heat source model that approximates an inverted cone (Figure 3), which is more in line with the actual heat source distribution.

In LTW, the distribution of laser energy in depth satisfies the Beer–Lambert law based on the Gaussian volume heat source model, and the distribution of volume heat source can be expressed as follows [49]:

(3) $$I\left(x,y\right)=\left\{\begin{array}{c} \;\;0;\;for\;transparent\;part\\ \alpha \cdot \frac{P_{0}}{\pi R^{2}}\cdot \exp\left(-\frac{x^{2}+y^{2}}{R^{2}}\right)\exp(-\alpha z);\;for\;absorbing\;part \end{array}\right.$$

where *R* corresponds to the radius of the light spot; 𝛼 is the material absorption coefficient, which is related to the absorbent content in the absorbing material; and *x*, *y*, and *z* are the coordinates of points in the volume heat source.

The temperature field simulation of laser contour welding based on the volumetric heat source model revealed that laser power has a positive effect on both temperature and melt pool size [40], while welding speed has a negative effect. An increase in beam diameter increases the weld width WW but decreases the weld temperature, depth of the transparent part (DT), and depth of the absorbing part (DA). The sensitivity of carbon black content to weld temperature, weld width WW, and depth of the transmitted bright portion is positive, while the sensitivity to DA is negative, and changes in carbon black content have a stronger effect on WW than on DT and DA. The deepest penetration of the weld is in the absorbing part [50]. By increasing the concentration (from 0.5–1.5%) of carbon black in the polymer, the laser power can be reduced to some extent. Composites with higher CB content (>1 wt%) are more difficult to weld due to the degradation temperature [19].

**Figure 4:**
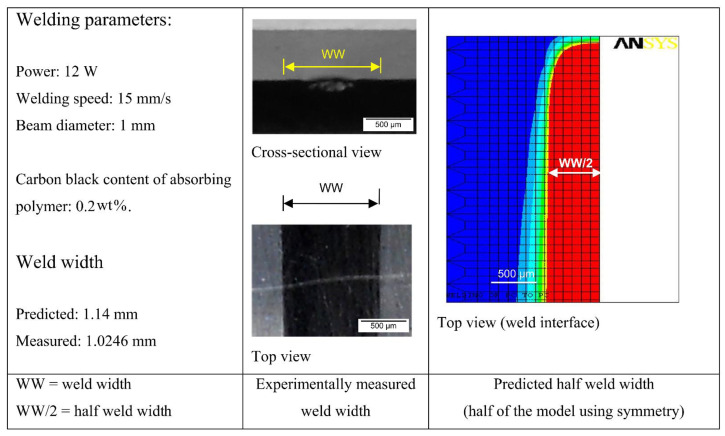
Comparison of the FE model’s predicted weld width result with the experimentally measured result [49]. (The color of the temperature field from red to blue indicates the temperature from high to low, and the red area represents the melted material. The figures of the temperature field that follow indicate the same meaning).

The transient temperature field of laser quasi-synchronous welding of PC (polycarbonate) based on a three-dimensional model simulation is shown in Figure 4. The simulation demonstrates that process variables such as laser power, welding speed, number of scans, and line energy have a significant effect on the temperature field during the welding process. The weld width is influenced by the combination of laser power, welding speed, and the number of scans. Increasing laser power and the number of scans result in wider welds while increasing welding speed reduces weld width. 

**Figure 5:**
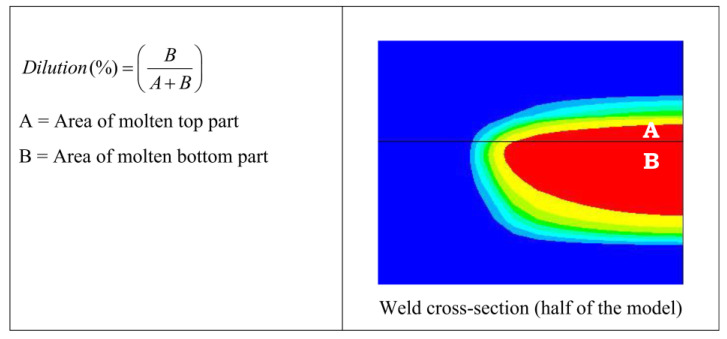
Schematic representation of the procedure to estimate dilution from the predicted weld pool [51].

**Figure 6:**
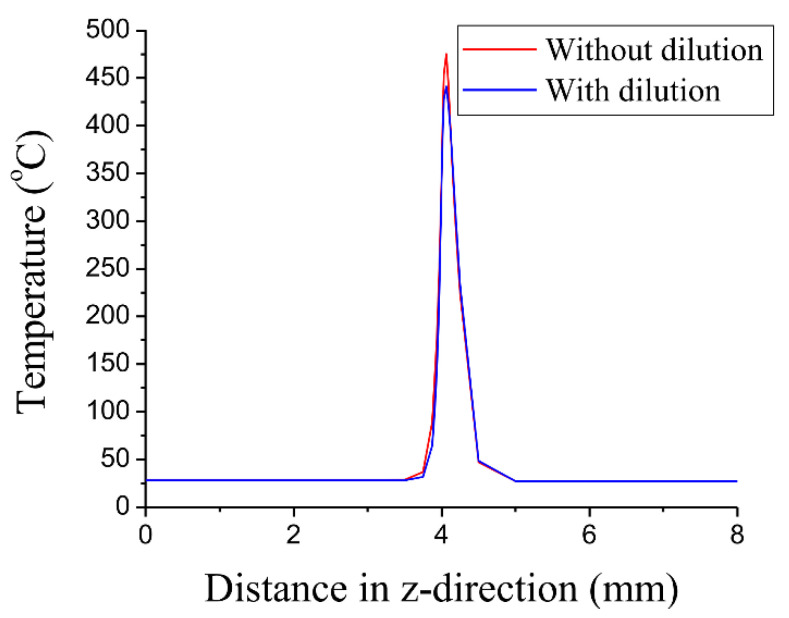
Temperature distribution at the weld interface along the z-direction [51].

A three-dimensional finite element model (FEM) of LTW between dissimilar plastics [51] considered the effect of melt pool dilution effects on temperature cycling for the first time. The welding temperature fields of PC (polycarbonate) and ABS (acrylonitrile butadiene styrene) were simulated using ANSYS and verified experimentally. The dilution determines the composition of the weld pool when the thermophysical properties and chemical composition of the joining materials are different (Figure 5). The material properties in the melt zone vary according to the ratio of the melts of the different materials, affecting the temperature field within the weld pool (Figure 6). The dilution effect mainly affects the highest temperature zone. Due to the difference in the glass transition temperatures of PC and ABS, the melt width at the weld interface is not the same for both. 

#### 2.2.1. Temperature Field Numerical Simulation Considering Interfacial Contact Conditions

The interface between the transparent layer and the absorbing layer is a rough point contact or local contact state in transmission welding simulation. Traditional models generally assume that the interface contact state is absolutely smooth without considering the real three-dimensional morphology of the contact surface or the effect of the contact surface gap between the actual welded specimens on the temperature field [52]. In the process of LTW, the presence of contact thermal resistance [53] leads to an obstruction of the heat transfer between the transmitted and absorbed layers, which in turn affects the distribution of the temperature field. 

**Figure 7:**
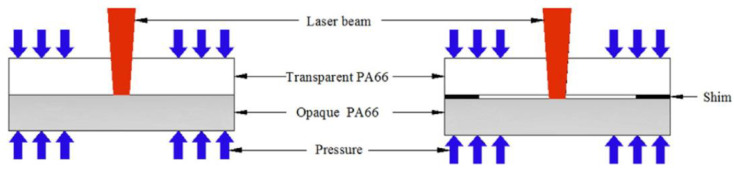
The schematic diagram of LTW under two kinds of interfacial contact status: (**left**)—without shim; (**right**)—with shim (nominal thickness is 20 μm) [54].

**Figure 8:**
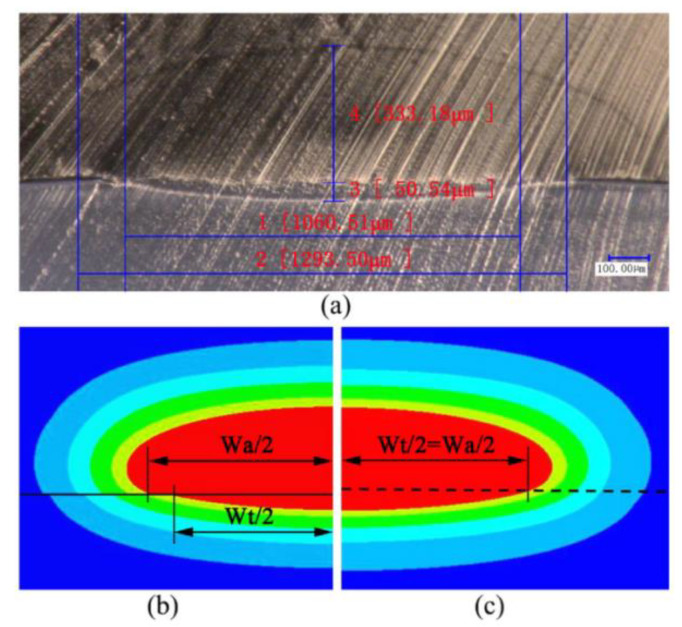
The profile of weld HAZ at the cross-section when P = 8 W and S = 0 for PA66: (**a**) HAZ of the experiment; (**b**) HAZ of the thermal contact model; and (**c**) HAZ of the traditional model [54].

The weld temperature field and weld profile of transparent and opaque PA66 under two conditions of no interfacial gap (S = 0) and interfacial gap (S = 20 μm) were analyzed by numerical simulation based on the volume heat source model and experimental comparison [54] (Figure 7). For the conventional model, the HAZ width is the same for transparent and opaque materials (W_t_ = W_a_). While the thermal contact model accounts for a width difference ΔW between the HAZ of the upper and lower layers (Figure 8), it is considered more consistent with the real model. The width and depth of the HAZ of transparent PA66 with S = 20 are smaller than S = 0. The thermal contact model can effectively predict the change in HAZ and improve the accuracy of the numerical simulation when the interface contact state changes.

**Figure 9:**
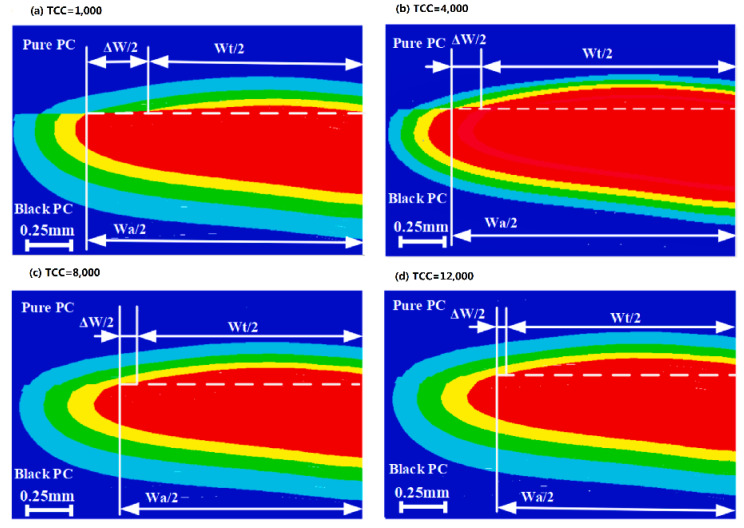
Temperature (°C) contours at the Y–Z plane under different TCC (W/(m^2^⋅K)) (Time = 0.5 s) [57].

**Figure 10:**
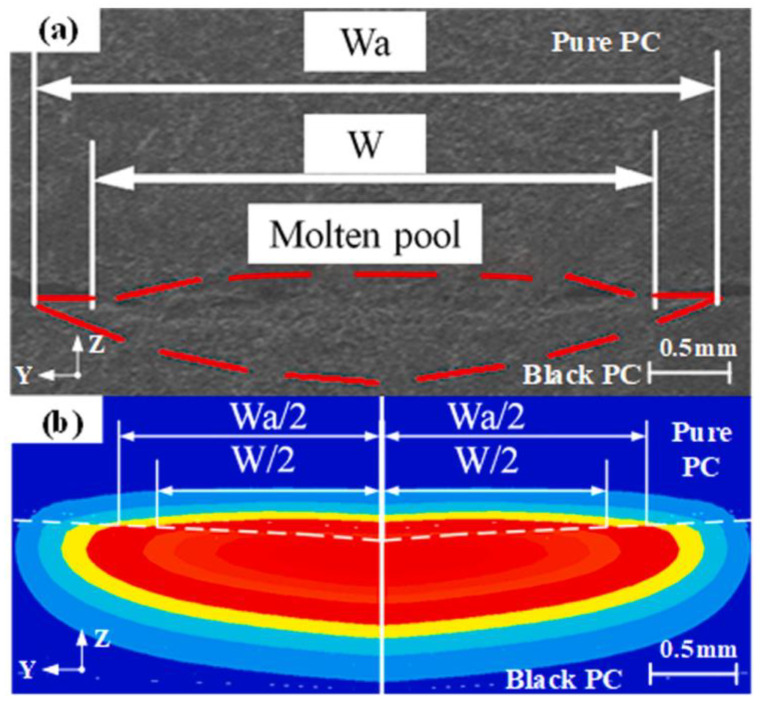
Weld width (P = 30 W, V = 15 mm/s, F = 0 MPa): (**a**) experimental weld width; (**b**) simulated weld width [57].

Thermal contact conductivity (TCC) is introduced into the thermal simulation of LTW to characterize the effect of the interfacial contact state on heat transfer [55].

(4) $$TCC=\frac{∆T}{q}$$

where *q* (W/m^2^) is the heat flux at the interface caused by the temperature difference (Δ*T*) to describe the heat transfer capability through the contact interface. When heat is transferred through a rough contact surface, gaps can lead to temperature discontinuities between the transparent part and the absorber layer, resulting in a smaller weld width in the transparent layer compared to the absorber layer [56]. These temperature discontinuities lead to temperature differences (Δ*T*) and secondary heat transfer at the interface. A contact model was developed to investigate the effect of TCC on the weld temperature field and weld seam dimensions of LTW [57]. Figure 9 shows the temperature field of the molten pool under numerical simulations of different TCC conditions. The maximum temperature in the weld decreases with increasing TCC. As TCC decreases, heat accumulates near the opaque material interface, and the contact state hinders heat transfer from the absorbing layer to the transparent layer. TCC affects the weld width and the width difference ΔW (ΔW = W_a_ − W_t_) between the upper and lower layers. TCC increases and ΔW decreases, which indicates that increasing TCC can improve the interface contact state and effectively improve the joint quality. TCC can be a key factor in the finite element model in LTW. The simulated weld width based on TCC agreed with the experimental weld width (Figure 10), verifying that the error of this prediction model was within 3.3%.

The relationship between the welding process parameters and TCC was derived using the response surface method [57]. The results showed that TCC increased with increasing laser power and decreasing welding speed. This could be due to the high laser energy input resulting in more material melting per unit time, leading to a shorter contact time of the lap surface, thereby reducing the effect of contact thermal resistance on heat distribution. TCC increases with increasing clamping pressure, and when the clamping pressure increases, the contact area at the interface of the two layers is conducive to heat transfer. The mathematical model was experimentally verified, and the error was within 5.5%.

#### 2.2.2. Numerical Simulation of Temperature Field for Laser Strategy Changes

The optical properties of the polymer (transmission, reflectance, and absorbance) are important parameters needed to simulate the LTW process since they determine the extent to which the laser energy in the polymer is absorbed and where it is absorbed. The optical properties of the polymer are wavelength-dependent [49].

**Figure 11:**
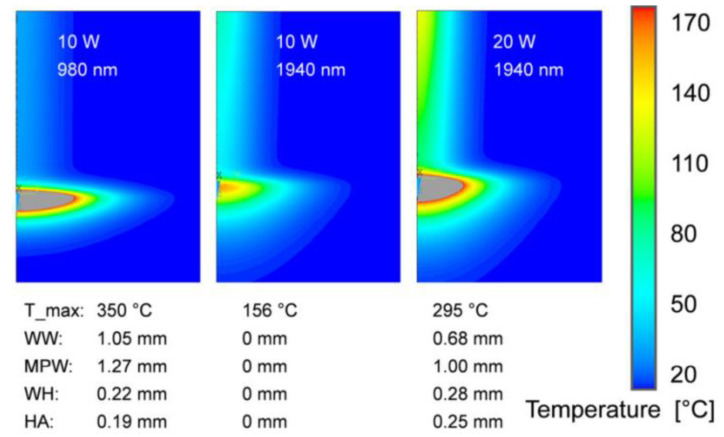
Calculated temperature fields and melt pool geometries for contour welding with different wavelengths [58].

**Figure 12:**
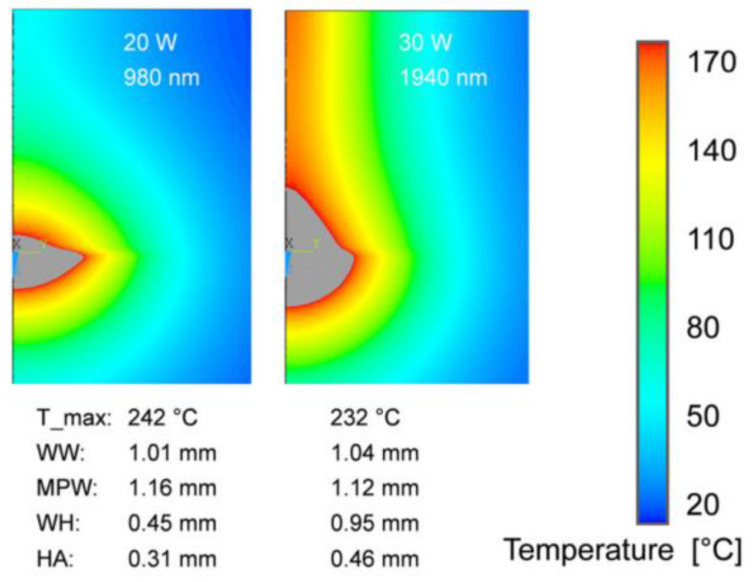
Calculated temperature fields and melt pool geometries for quasi-simultaneous welding with different wavelengths [58].

The temperature field was simulated for simultaneous and contoured welds and gaps, considering absorption and scattering properties, based on the volume heat source model at different wavelengths [58] (Figure 11 and Figure 12). The simulation results showed that the polymer had better absorption properties in the wavelength range of 1400–2000 nm compared to 980 nm. This allowed for deeper penetration of absorbing parts containing absorbers, resulting in a larger weld size, better inclusion of gaps, and reduced risk of degradation, ultimately improving the weld strength.

A numerical simulation of the temperature field can reveal the relationship between temperature, Rayleigh length, and absorption coefficient [59]. The absorption coefficient decreases with increasing wavelength, and the size of the heat-affected zone decreases with decreasing absorption coefficient and increasing welding speed. Laser welding of transparent materials without absorbers is achieved by controlling the distribution of the temperature field through proper laser beam focusing and wavelength adjustment. Nguyen et al. [52] proposed a computational model for the temperature distribution of quasi-synchronous welding without absorbers for a fiber laser wavelength of 1940 nm based on medical and biotechnological applications using a volumetric Gaussian heat source. The results showed the predominance of diffusive heat transfer in quasi-synchronous welding. The simulated HAZ height deviated from the experimental HAZ height by an average of 12.8%, and the simulated HAZ width deviated from the experimental HAZ width by a large 48.5%.

**Figure 13:**
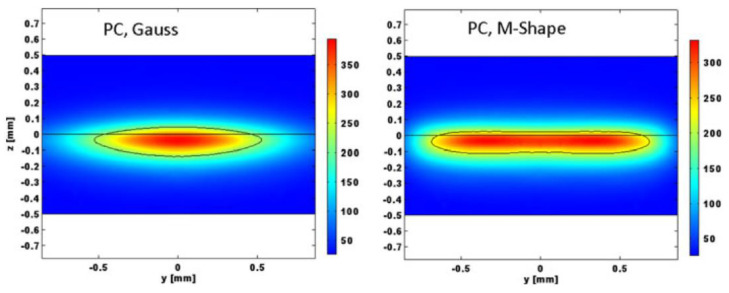
Temperature field (°C) in a (y, z) −plane section for PC, Gaussian distribution (**left**), M−shape (**right**), power: 5 W, radius: 850 μm, velocity: 25 mm/s, 220 °C isotherm. The intensity values are taken from the top (transparent part) and bottom (absorbing part) of the figure [60].

**Figure 14:**
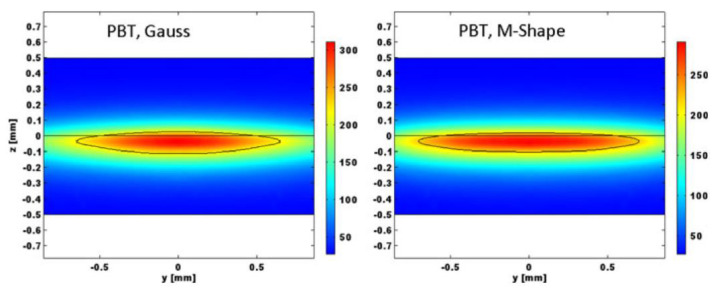
Temperature field (°C) in a (y, z)—plane section for PBT, Gaussian distribution (**left**), M—shape (**right**), power: 18 W, radius: 850 μm, velocity: 25 mm/s, 220 °C isotherm. The intensity values are taken from the top (transparent part) and bottom (absorbing part) of the figure [60].

Finite element simulations were performed based on the volume heat source to calculate the different temperature fields generated by Gaussian and m-shaped beams, respectively [60]. The simulations were conducted for LTW of PC, a non-scattering polymer, and PBT, a strongly scattering polymer. Figure 13 and Figure 14 show the results of the simulations. For PC, the m-shaped beam produces a joint with constant thickness and width, which is proportional to the platform width of the integrated intensity. For Gaussian beams, the thickness is not constant, and the width is not directly proportional to the beam diameter. Gaussian beams lead to higher temperatures and a higher probability of degradation. For PBT, the volume scattering destroys the original beam shape, and the difference in the beam distribution in the connection zone is flattened by the scattering process in the transparent part. As a result, the dimensions of the model joints of the two beams are similar.

Numerical simulations allow obtaining the temperature field of multiple heat sources superimposed. Geißler [61] established a finite element model using the sum of two bulk heat source models superimposed to simulate the temperature field of a diode laser (λ = 920–1050 nm) and a fiber laser (λ = 1940 nm) used to increase the energy absorption effect of the upper partners. The study investigated the effect of the two superimposed laser sources on the temperature field and weld geometry during both contour welding and quasi-synchronous welding processes. Results showed an increase in the size of the heat-affected zone of the upper part, which could potentially lead to reduced residual stresses and increased gap-bridging capability. In contrast to the heat-affected zone of contour welding, in the case of quasi-synchronous welding, a rather teardrop-shaped heat-affected zone was formed. In addition, the additional laser radiation led to an increase in the weld depth ratio for uniform heating of the upper joint partner. The average deviation of the results of this model from the experimental values was between 8.5% and 27.4% for all weld geometry features.

### 2.3. Temperature Field Simulation Based on Combined Heat Source Model

The volume-distributed heat source considers the distribution of the heat source in the direction of the melting depth. However, it simplifies the heat source distribution law by assuming that it is the same for the surface and the interior of the melt pool. In fact, there are some differences in the heat source distribution between the melt pool surface and the melt pool interior. A combined heat source model is used to divide the total energy input into two parts and select a suitable heat source model for each part based on their different heat flow distribution characteristics. The combined heat source model for laser welding numerical simulation has also been studied and verified [62]. The study of LTW of PC sheets by Goyal et al. compared the surface temperatures obtained from the developed numerical model with volume and surface heat fluxes with the surface heat source model and found that the former estimates of temperature were closer to the experimental results [63].

**Figure 15:**
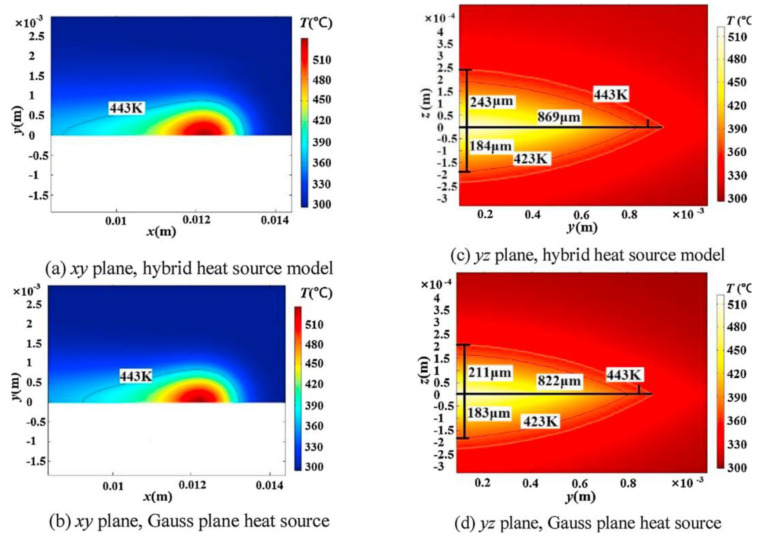
The temperature distribution of a half−molten pool is Q = 100 W, v = 100 mm/s, and P = +7 mm [64].

**Figure 16:**
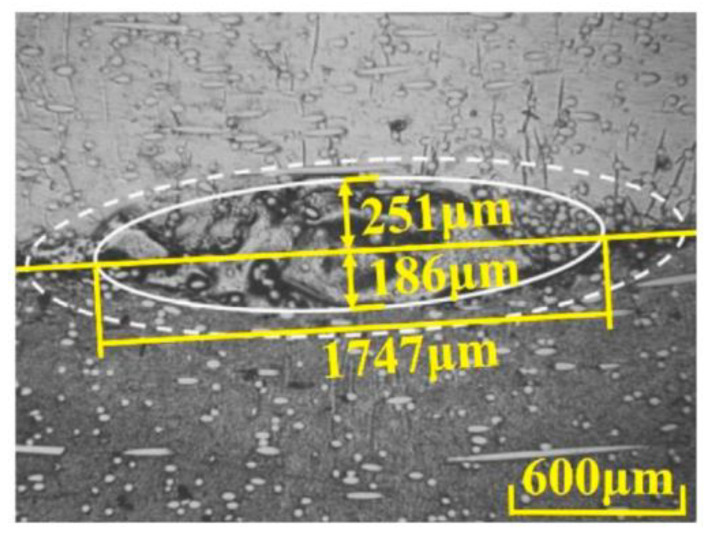
The microstructure of a molten pool is Q = 100 W, v = 100 mm/s, and P = +7 mm [64].

Chen’s study verified that the hybrid heat source model is more suitable for laser welding simulations of fiber-doped plastics than the single heat source model (volumetric or planar Gaussian heat source) [64]. Chen established a hybrid heat source model that combines a rotating Gaussian volume heat source and a Gaussian planar heat source for LTW of glass fiber-reinforced plastics. This means that a rotating Gaussian volume heat source is used to describe the heat flow density of the transparent portion of the laser, and a planar Gaussian heat source is used to represent the heat flow density of absorber-absorbed energy. By comparison, it was found that the hybrid heat source agreed better with the experimental data than the Gaussian planar heat source. Figure 15 shows the temperature distribution of the mixed heat source model and the planar heat source model. Figure 16 shows that the average width of the hybrid heat source simulation is 1738 μm, and the melt pool thicknesses of the PP and ABS are 243 μm and 184 μm, respectively. The experimentally measured average width of the melt pool is 1747 μm, and the thicknesses of the PP and ABS melt pools are 251 μm and 186 μm, respectively. The temperatures predicted by the mixed heat source model and the surface heat source model are 270.4 °C and 264 °C, respectively, and the actual measured value is 271.2 °C. The results of the mixed heat source are closer to the measured data. Mathematical relationships between melt pool area, laser energy per unit area, and shear strength were also established.

### 2.4. Temperature Field Simulation by Other Methods 

In addition to the above studies of temperature fields based on various types of heat source models, some researchers have used quasi-static models, ray tracing, and other methods for temperature field studies.

The quasi-static model introduces mass flow in the model, a time-dependent contour welding process, as a time-independent heat transfer problem (quasi-static model) to solve. The method was used to simulate the heat transfer process in amorphous polymer-polycarbonate (PC) contour laser transfer welding [65], and the temperature fields were obtained for different combinations of laser scan power and speed. The simulation results were consistent with the experimental data. The convergence time was short (within 3 min for a set of process parameter conditions).

The scattering properties of the material in LTW influence the absorption of welding energy [66]. The laser beam is scattered by internal reflection and refraction when the laser is incident on the material, reducing the amount of radiation available for melting the base material in the weld zone. When thermoplastics are highly crystalline polymers or filled with materials such as reinforcing fibers or colorant titanium dioxide, the connected parts produce more scattering, which negatively affects the welding process. To obtain accurate simulation results for the temperature field, a numerical model considering light scattering is required. The laser intensity distribution after passing through the upper scatterer in transmission welding is first determined, and then finite element simulations are performed. Experimentation is one method to determine the laser intensity distribution, which involves using a CMOS camera sensor [67] or a video camera to monitor the laser intensity distribution after passing through the upper material [68]. The determined intensity distribution is then quantified with respect to the original laser beam [69], and a Gaussian function is used to fit the intensity distribution for the temperature field finite element simulation. The intensity distribution of the heat source considering light scattering is wider in width and lower in peak height than that of the heat source not considering light scattering. In particular, the difference in intensity distribution is more pronounced when the transparent part of the laser contains enhanced material than when the transparent part of the laser is not enhanced.

**Figure 17:**
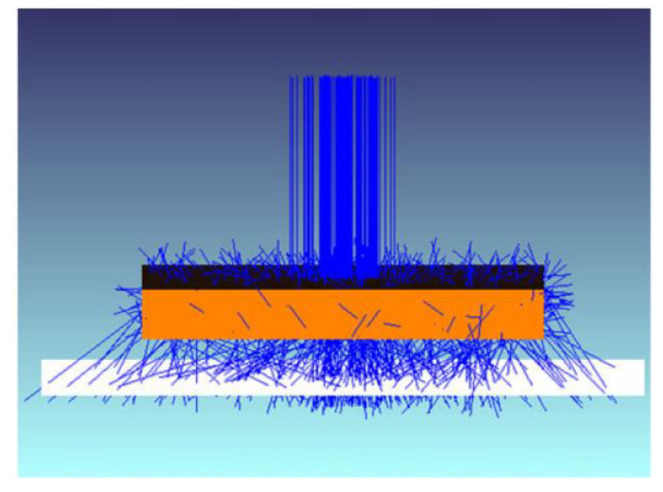
ZEMAX™ scheme, top box: transparent part, white plane: simulation detector (distance to part is enlarged for better visualization), lines: rays of radiation (incoming and scattered) [60].

Another method is the ray-tracing method. The ray-tracing method is a simulation of separating the incident laser beam into N microbeams from a laser head and line propagation based on the changing direction of the laser through the medium, as shown in Figure 17. Simulation involves modeling the geometry of the laser beam and the resulting temperature field that propagates through the material. This method allows the simulation and optimization of the laser source as well as provides a good estimate of the laser beam intensity distribution at the weld interface [70]. The intensity distribution below the transparent part was simulated using ray tracing [60]. To investigate the effect of two absorbers, carbon black and ITO, on the heat-affected zone [17], ray-tracing software ZEMAX was used to simulate and calculate the power density normalized to 1 W input power. The power density obtained from the simulation was used for the calculation of the finite element temperature field. The thermal simulation results showed that the laser power of TiO had to be increased by an order of magnitude to reach the same maximum temperature compared to CB. 

**Figure 18:**
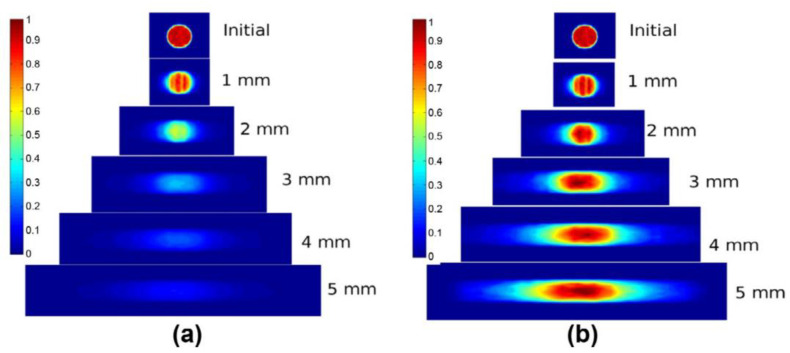
Resulting laser beam energy profile through composite substrate thickness (**a**) with respect to initial power and (**b**) with respect to the maximum power [71].

The ray-tracing method can be used for the numerical simulation of laser welding of thermoplastic composites [71]. The microstructural heterogeneity of the material in terms of optical and thermal properties was considered to simulate the optical path of the laser beam through the composite structure. The study also analyzed the influence of the fiber structure on the welding process and showed a nonlinear relationship between the welding energy and the thickness of the substrate (Figure 18). Additionally, important parameters for optimizing the welding process were identified.

The ray-tracing method was combined with the finite element method to simulate the temperature field during LTW of glass fiber-reinforced thermoplastic composites [72]. Ray-tracing software was used to calculate the heat source terms used in the finite element simulations and was able to deal with absorption and strong light scattering caused by the non-uniformity of the polymer matrix and glass fibers.

## 3. Stress Simulation for Laser Transmission Welding

During the LTW process, the laser locally heats the material, causing a significant temperature increase, resulting in local thermal expansion and contraction. The deformation is constrained by internal stresses, and the material relaxes after the weld cools due to its thermal viscoelasticity. The residual stresses are then formed by the unrelaxed thermal stresses within the welded parts. Welding residual stresses can lead to cracks and deformation [73,74]. Moreover, the size and distribution of these stresses can significantly affect the behavior of the welded component during its service life, resulting in reduced strength and a shortened service life. In particular, tensile stresses can adversely affect the local mechanical properties of welded components [26]. 

The formation and development of welding strains and stresses are related to several factors, such as clamping conditions, thermomechanical material properties, type of welding process, process parameters, ambient temperature, and cooling conditions. Numerical simulations were used to establish a thermomechanical model to analyze the welded thermal stress field and predict the distribution and magnitude of stresses and residual stresses in the LTW process. The mechanical behavior in welding is calculated and simulated to obtain the appropriate process parameters to minimize the residual stresses. The stress–strain model was developed through the theory of thermoelasticity and plasticity [75], in which the overall strain consists of the elastomeric, plastic, and thermal components. The errors between the simulated residual stresses and the experimental results are due to the simplification made in the simulation model. For instance, the decomposition of the material in the experiment may have been ignored, and the interface roughness and variation of the material parameters with temperature may not have been considered. Additionally, the limitations of the residual stress measurement method may also have contributed to the errors.

A nonlinear transient thermo-mechanical finite element model of the LTW of PPS was developed using the optimized set of process parameters [76]. The residual stress was calculated to be 14 MPA at 320 °C. Temperature measurements in the experiment were conducted by thermocouples, and no experimental verification of the stress magnitude was performed. 

Considering the asymmetric compression-tension behavior of the material [77], the instantaneous equations for the stress–strain variation of the material with time were established. Moreover, the effect of the process parameters on the residual stress behavior during the heating and cooling stages was simulated using a finite element model. Tensile residual stresses of 25 MPa were calculated using an elastic viscoplastic material model of polycarbonate (PC) up to 250 °C after cooling in the transport direction. Hopmann [78] established a thermodynamic model to calculate the residual stress in PA 6.6 within the range of 15–25 MPa. Based on this model, another model was established to predict the strength of the weld, which was verified through tensile testing.

The formation and development of welding strains and stresses are related to several very closely related factors, such as clamping conditions, thermo-mechanical material properties, type of welding process, process parameters, ambient temperature, and cooling conditions. 

Based on the finite element simulations of the temperature field of nylon 6, the relationships between weld width, melt penetration, residual stresses, and process parameters were determined [26]. By using a multilinear isentropic hardening model integrating the von Mises yield criterion to represent the residual stress–strain behavior, the optimal parameter conditions for thermomechanical analysis of nylon 6 were determined. The results showed that the minimum residual stress = 1,487,793 N/m^2^, and the optimal parameters were obtained through simulation. The residual stress in the material increases with increasing power and decreases with increasing scan speed and beam diameter. The increase in residual stress is due to the greater deformation of the material relative to the power increment.

**Figure 19:**
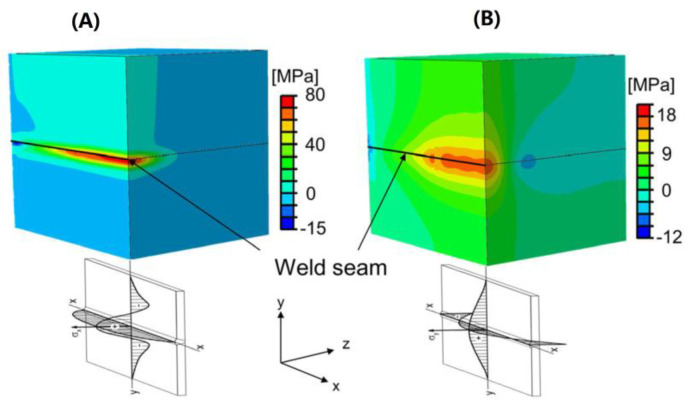
Normal stresses of the weld seam (**A**) in Longitudinal direction (x−direction); (**B**) in transverse directions (y−direction) [79].

A thermomechanical model based on the temperature field was established to analyze the factors associated with the generation of residual stresses. These factors include thermal expansion, temperature-based phase changes, and stress-induced phase changes [79]. The model was used to calculate the distribution of residual stresses induced by the welding process based on the temperature-dependent behavior of the elastic material. The numerical simulation results are shown in Figure 19, where both normal stress directions are in good agreement with the theoretical stress profile. The colder region away from the joint prevents the inner region from shrinking, which generates tensile stresses (−σ_x_), and the colder region generates compressive stresses (−σ_x_). The lateral shrinkage of the weld also generates tensile stresses (+σ_y_), but because the compressive stress at the end of the equilibrium weld is (+σ_y_).

**Figure 20:**
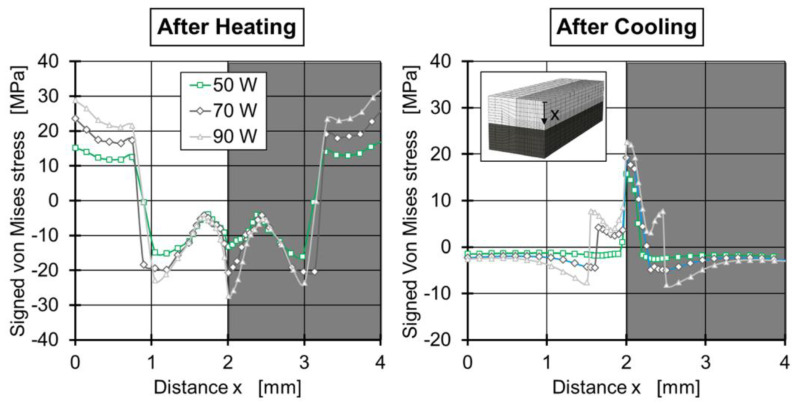
Spatial signed Von Mises stresses for the elastoplastic material model with laser powers of 50 W, 70 W, and 90 W after heating and cooling [80].

A thermodynamic model with elastic, plastic, and viscoelastic material properties was developed for PA 66 [80]. The stresses and strains due to thermal expansion and the temperature gradients during heating and cooling in the welding process were calculated. The heat-affected zone is obtained by accurate experimentation of the thermal properties and a newly developed extinction coefficient measurement device. As shown in Figure 20, as the laser power increases, the tensile and compressive stresses also increase, and the maximum tensile stress is located in the center of the weld. The calculated residual stress exceeded the yield point of the polyamide only an elastoplastic material model could actually calculate the tensile–compression ratio of the thermally induced residual stresses during the welding process. The residual stress values were verified using the drilling method. However, the effective depth of the measured material was only 0.6–0.8 mm. As a result, the calculated residual stress at a depth of 2 mm in the weld seam could not be verified quantitatively.

**Figure 21:**
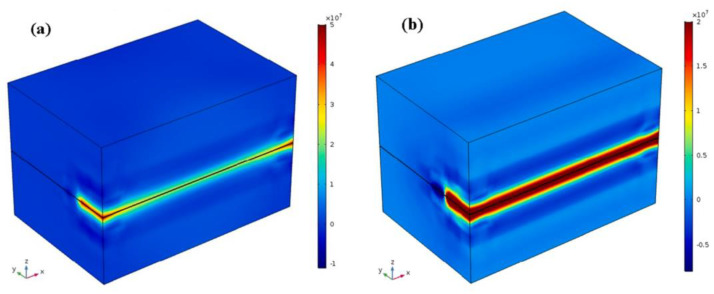
The cloud chart of the residual stress distribution, (**a**) the longitudinal stress σ_x_ distribution diagram, (**b**) the transverse stress σ_y_ distribution diagram [81].

Numerical simulations of the stress scenario with the metal film as the absorber were carried out. A three-dimensional thermomechanical coupling model of residual stress distribution in LTW of polycarbonate (PC) based on copper film interlayers (PC/Cu/PC) was developed by applying COMSOL [81]. The residual stress distribution is shown in Figure 21, with tensile stresses in the middle of the weld and compressive stresses in the vicinity of the weld. Dynamic and static strain gauge test systems were used to evaluate the residual stresses. The results showed that both simulated and experimental residual stresses increased with increasing welding power and copper film width, while the effect of welding speed was reversed. The maximum deviation between the simulated residual stresses and the experimental residual stresses was 1.2 MPA, which indicated that the simulation and experiment were in good agreement. The thermal expansion of the PC was 30 times higher than that of the copper film, and the expansion of the PC had a more significant effect on the thermal stress, which led to multiple fluctuations forming at the weld seam around the laser spot [82].

The evolution of thermodynamic and thermomechanically induced stress fields in the heating and cooling phases of polymer laser transmission welding was investigated numerically [83]. A 3D transient thermodynamic model was designed to simulate laser transmission contour welding with a moving laser beam. The temperature results of the thermal model were added to the relevant mechanical model using a sequentially coupled field analysis. A multilinear isotropic hardening model was used, which incorporates the von Mises yield criterion, the combined growth rule, and the isotropic hardening law. The viscoplastic effects of the polymer were incorporated by implementing Perzyna’s rate-dependent plasticity model in ANSYS. The model was able to predict transient temperature and stress fields and residual stresses. The stress distribution pattern for laser-welded polycarbonate obtained from this simulation is similar to the temperature distribution, with the maximum stress occurring just behind the beam position. Higher stress concentrations occur in the adjacent area around the weld pool, and compressive stresses are gradually converted to tensile stresses after subsequent cooling.

## 4. Melt Flow Field Simulation for Laser Transmission Welding

Numerical simulation of the LTW temperature field has been extensively studied, but not much research has been conducted on the melting behavior, fluid flow, and related convective heat aspects of the welding process. The simulation of the flow field of the melt is inextricably linked to the simulation of the temperature field. The change in temperature affects the amount of material melted, and the pressure and temperature between the interfaces determine the fluid flow state. The fluid flow of LTW is numerically modeled to predict the geometry and porosity formation of the weld seam. 

**Figure 22:**
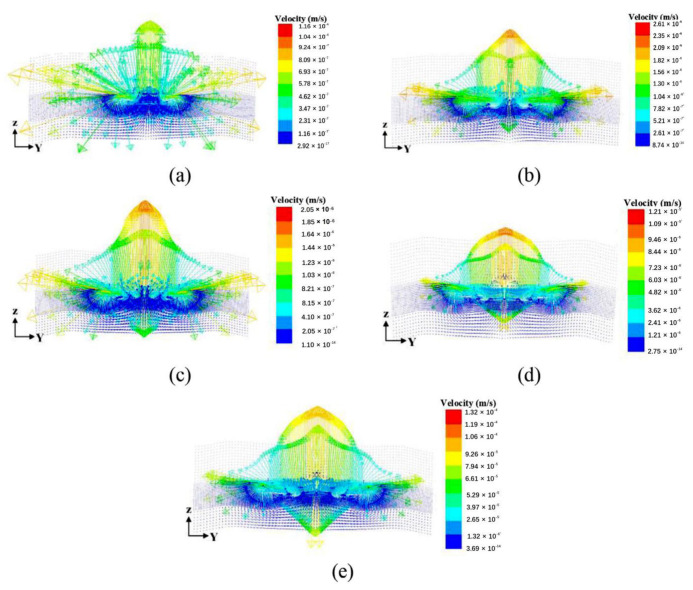
Flow field variation with laser power: (**a**) P = 20 W; (**b**) P = 30 W; (**c**) P = 40 W; (**d**) P = 50 W; and (**e**) P = 60 W [56].

Numerical simulation results and experimental verification of the flow field of LTW show that the welding process parameters have a significant effect on the fluid flow, which ultimately affects the heat transfer, the geometry of the melt pool, and the formation of the weld seam. As the laser power increases, the fluid velocity in the molten pool also increases. When the welding speed decreases, more energy is absorbed, resulting in a higher maximum temperature. This increase in temperature causes a decrease in viscosity, leading to a higher fluid flow intensity. Consequently, the weld width and height also increase. Moreover, the heat absorbed between the interfaces is carried by the circulating fluid to the edge of the molten pool, which greatly expands the pool’s width. The maximum flow velocity of the melt is proportional to the increase in clamping force. As the clamping force increases, the actual contact area of the two contact surfaces also increases, leading to an increase in heat transfer [56]. Figure 22 shows the ANSYS simulation of the flow field variation at a welding speed of 15 m/s with a clamping force of 0.12 MPa and different laser powers.

**Figure 23:**
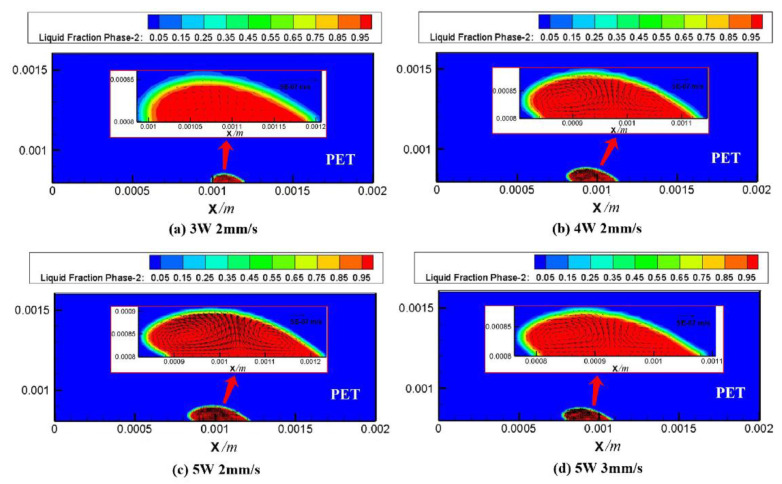
The geometry of the molten pool and velocity field: (**a**) Case 1; (**b**) Case 2; (**c**) Case 3; (**d**) Case 4 [84].

**Figure 24:**
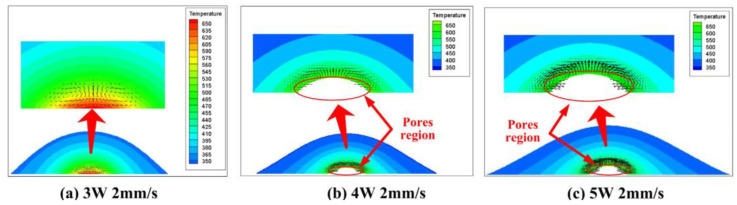
The calculated bubble regions for different welding conditions: (**a**) Case 1; (**b**) Case 2; (**c**) Case 3 [84].

Ai et al. [84] developed a 3D transient numerical model for the LTW of the PET/titanium alloy Ti6Al4V, considering melting and fluid flow. The temperature field, melt pool, and fluid flow were simulated for different laser powers and welding speeds. The simulation predictions of the weld geometry are in good agreement with the experimental data. The results show that the trend of the weld geometry is closely related to the flow pattern and flow rate in the molten pool, which is expanded to an elliptical shape as shown in Figure 23. Porosity persists in the high-temperature region due to the high-temperature decomposition of PET in the center of the weld (Figure 24).

For the study of the relationship between melting behavior and heat transfer during laser transfer welding, a three-dimensional transient model based on the finite volume method was established to simulate the formation of the melt pool during the laser transfer of PET and SUS304 [85]. Simulation results show that the melt pool is slow and laminar, and the flow in the melt pool is characterized by vortex flow due to the local heating and processing characteristics of laser welding. As the heat input increases, the maximum velocity in the melt pool increases, and the size of the melt pool increases. The effect of heat conduction is more pronounced than the effect of convection. Therefore, the temperature at the interface is the key to melt pool formation. Additionally, the size of the melt pool in the direction of heat source movement depends mainly on the heat input and the rate of temperature drop.

A mathematical model of transparent acrylate and polycarbonate laser oscillation welding [25] was developed to study the effect of beam oscillation on the process response. Beam oscillation produced uniform heat distribution and turbulence within the weld pool, which improved material mixing and joint strength. Morphological analysis showed the presence of some tiny bubbles on the top surface of the weld, which enhanced the micromechanical connections at the weld interface. An artificial intelligence-based teaching learning optimization (TLBO) algorithm and a desired function analysis (DFA) based optimization method were used to improve the weld quality and obtain the desired response. TLBO produced more accurate results than DFA. The circular beam oscillation with an oscillation diameter of 0.6 mm and a frequency of 3 kHz had a substantial beneficial effect on the weld seam strength.

**Figure 25:**
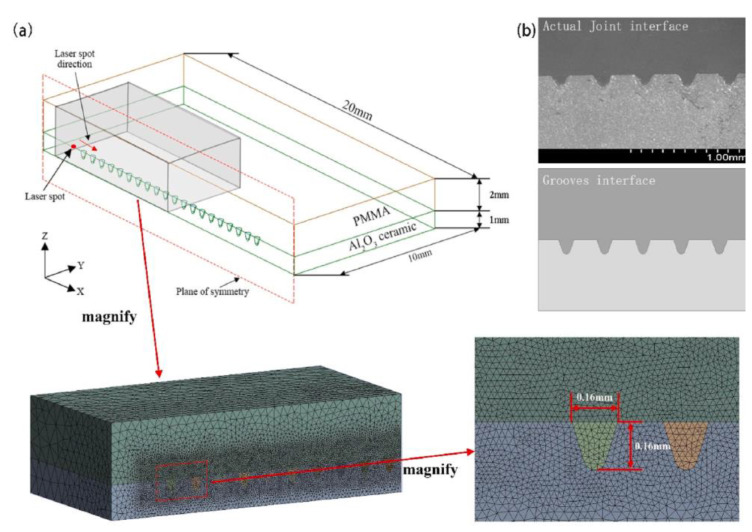
(**a**) Schematic diagram of the model and coordinate system adopted in calculation and meshing; (**b**) the actual joint interface and model used in simulation [86].

**Figure 26:**
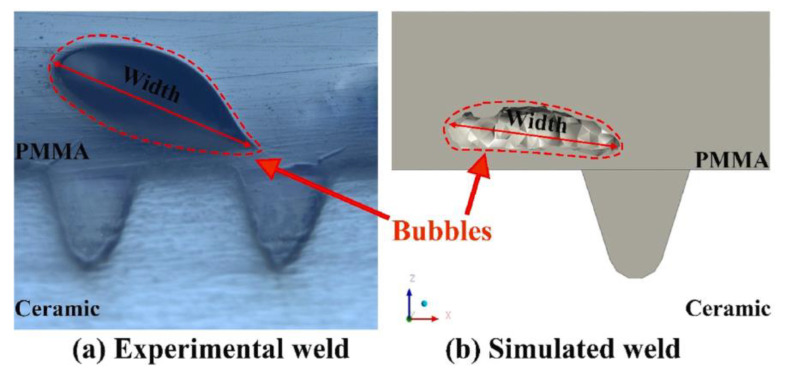
The bubble size comparison between simulation results and experimental results. (**a**) Experimental weld and (**b**) simulated weld [86].

**Figure 27:**
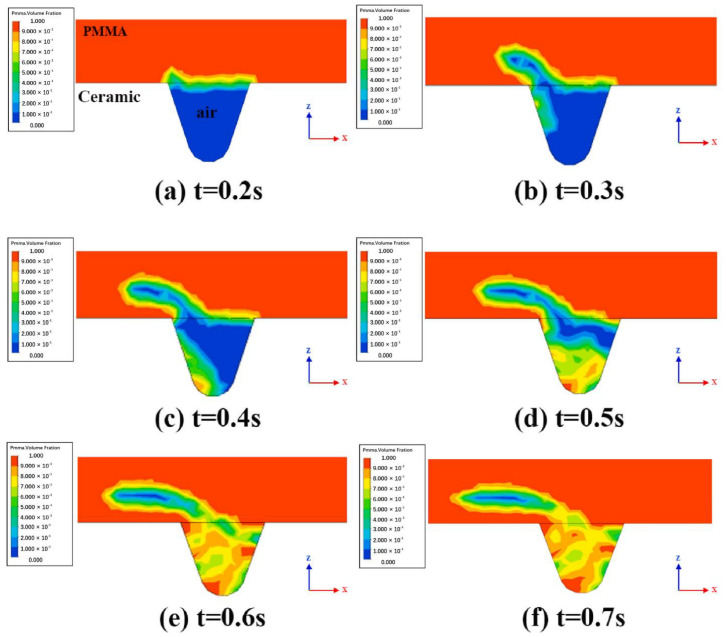
Schematic diagram of volume distribution in polymer flow (y = 0 mm, P = 80 W, v = 2 mm/s, d = 0.9 mm) [86].

The temperature and flow fields during the welding of highly transparent polymer/alumina ceramics were simulated based on the volume heat source model [86]. The grid model is shown in Figure 25. It explored the effects of blind-hole microtexture size, laser power, and laser scan rate on solution flow and weld strength and revealed the flow of polymer melt, bubble formation, and melt pool shape formation. The results show that the blind-hole microtexture can effectively increase the laser energy absorption rate of the ceramic material and promote the polymer to enter the blind-hole microtexture, forming a mechanical riveting joint, which is the main factor for achieving a high-strength joint. The bubbles in the weld all appeared in the upper left corner of the blind-hole microtexture (Figure 26). The position of bubble formation in the weld was consistent with the simulation, and the size of the weld also matched the simulation results. Figure 27 illustrates the process and principle of the polymer melt being pushed into the blind pore microstructure by the expansion gas compression.

## 5. Thermal Degradation Simulation in LTW

The thermal degradation of the material has a significant effect on the weld strength of LTW. Both excessively high laser power and excessively low laser scanning speed can result in elevated welding temperatures, leading to polymer degradation and void formation at the weld interface, ultimately causing a significant reduction in weld strength.

Thermal degradation is a kinetic phenomenon that depends on both temperature and time. When the temperature exceeds the degradation temperature, the material does not immediately decompose and needs to be retained at the degradation temperature for a certain period. The retention time of the polymer at the decomposition temperature decreases exponentially with increasing temperature [68]. The experimental methods can only predict the range of process parameters at which material degradation begins, and it is difficult to determine the amount of material degradation for different process parameters. However, numerical simulations can be used to make such predictions [87]. The thermodynamic model of pyrolysis obtained by thermal gravimetry analysis was combined with temperature–time data from numerical simulations to predict the amount of thermal degradation of the polymer during LTW. Thermal gravimetry analysis (TGA) is an experimental technique commonly used to study the kinetics of material pyrolysis reactions by measuring the weight change of polymer samples as they are heated and calculating kinetic parameters [88]. Khosravi [89] used kinetic data obtained from a single thermal rate TGA study and temperature–time distributions obtained from a two-dimensional finite element thermal model to predict the thermal degradation of blends of PBT and PET. The predictions made by the model were within 10–20% error from the experimental values. It was also found that increasing the proportion of PET in the blends resulted in more thermal degradation, and the blends containing PET achieved higher weld strength than pure PBT. The SB model, the nth-order model, and the 3D quasi-static finite element thermal model were combined by Bates et al. [90] to simulate the thermal degradation of PC and PA6 in LTW. The study found that the power at which degradation begins decreases with increasing absorber content, which is consistent with the power at which shear strength begins to decrease. Wang et al. predicted the thermal degradation in the welding of black and natural polyamide 66 (PA66) by combining a pyrolysis kinetic model and a three-dimensional transient thermal model of the volumetric heat source [84]. The kinetic parameters of PA66 were obtained based on TGA data using an n-order model in MATLAB. Using this kinetic response model containing the relevant kinetic parameters, the temperature–time data at the highest temperature point with different weld parameters were predicted. Additionally, the variation of the material conversion rate with temperature (time) was analyzed. It was found that the predicted power at which the material starts to degrade is in general agreement with the power at which the shear strength starts to decrease.

## 6. Summary

**Figure 28:**
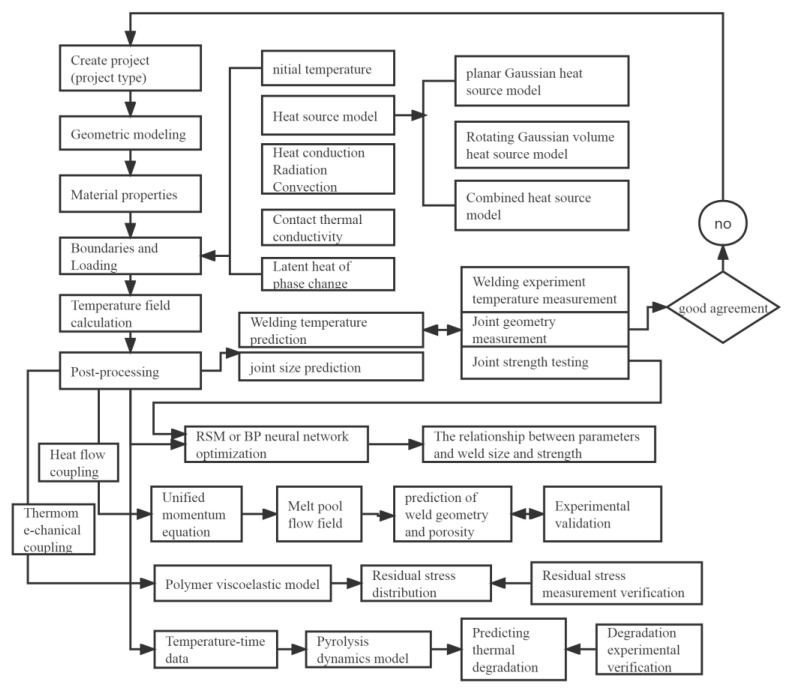
General view of LTW numerical simulation analysis.

Numerical simulation is an important research method and field for studying LTW processes and mechanisms. Figure 28 shows a general view of the numerical simulation system for LTW. The corresponding models are established for different welding materials and different processes. Moreover, numerical simulations of the temperature field, stress field, and melt flow field acting individually or coupled with each other and thermal degradation are performed. For simulations, an appropriate heat source model is selected based on the actual welding conditions, such as a surface heat source, a volumetric heat source model, a combined heat source model, or others. Multiple factors are taken into account in the simulation, including density, thermal conductivity, specific heat capacity with temperature, the roughness of the contact surface, properties of the absorber, wavelength, etc. The simulation results should be experimentally verified and calibrated.

The numerical simulations of LTW provide several insights. Firstly, they help obtain the distribution of the LTW temperature field and study the effect of parameters such as laser power and welding speed on the temperature field. These simulations also derive the law between the parameters and joint performance, predict the maximum temperature under a given parameter, and obtain the shape of the heat-affected zone, the width and depth of the melt pool, and optimal process parameters for LTW. Secondly, they study the contact thermal resistance model considering the interface roughness and contact condition and obtain the effect of contact surface roughness on the temperature field and weld quality, thus improving the accuracy of numerical simulation. Thirdly, they investigate the influence of absorber type, content, and distribution on the temperature distribution and weld profile during LTW and obtain the optimal parameters of the absorber. Fourthly, some new processes, such as wavelength changes and heat source superposition are studied by simulations. Fifthly, these simulations help obtain the weld stress field and residual stresses, and predict post-weld deformation and defects. Sixthly, the formation of melt pools, fluid flow, and porosity are investigated. Seventhly, the degradation of the material during welding is predicted by combining the pyrokinetic model and temperature–time simulations.

## 7. Conclusions and Prospect

This paper describes the numerical simulation of LTW, covering the simulation methods used in the temperature field, flow field, stress field, and degradation prediction, the simulation results and validation, and the application of numerical simulation. The following are further scopes for LTW simulation in the future: (1) High-fidelity models and fast simulation methods, as well as the integration of other mathematical methods, should be explored in the future study of LTW. In LTW simulation, high-fidelity models can more accurately and effectively simulate the welding process, visualize the trend in weld quality as process parameters change, and thus guide the production of the process. (2) Explore easier-to-implement and more accurate means of measurement. Numerical simulations still need to be validated and calibrated based on existing experimental results. The accuracy of the model and simulation process can only be very close to reality. However, there will still be some discrepancies. Existing experimental validation methods, such as temperature field measurements and stress measurements, still have limitations. Test methods for welding temperature fields, process stresses, and residual stresses cannot be measured accurately. (3) Simulation studies can be further applied to LTW between various high-performance composite materials, thermoplastic materials, metal materials, and other dissimilar materials, as well as to explore new welding processes and further investigate the welding mechanism and process parameters.

## Data Availability

Not applicable.

## Abbreviations

LTW	Laser transmission welding
WW	Weld width, and
DA	Depth of penetration in absorbing material
HAZ	Heat affected zone
FEM	Finite element mode
CB	Carbon Black
PET	Polyethylene terephthalate
PC	Polycarbonate
PBT	Poly (butylene terephthalate)
ABS	Acrylonitrile butadiene styrene
PEEK	Poly-ether-ether-ketone
DFA	Desired function analysis
TLBO	Teaching-learning-based optimization
PA 66	Polyamide
RSM	Response surface methodology
TCC	Thermal contact conductivity
ANN	Artificial neuronal networks
NSGA-II	Non-dominated sorting genetic algorithm II
TGA	Thermal gravimetry analysis
